# What Protects Adolescents with Youth Subculture Affiliation from Excessive Internet Use?

**DOI:** 10.3390/ijerph15112451

**Published:** 2018-11-03

**Authors:** Daniela Filakovska Bobakova, Jana Holubcikova, Andrea Madarasova Geckova, Zuzana Dankulincova Veselska

**Affiliations:** 1 Department of Health Psychology, Faculty of Medicine, P.J. Safarik University in Kosice, Tr. SNP 1, Kosice 040 66, Slovak; jana.holubcikova@upjs.sk (J.H.); andrea.geckova@upjs.sk (A.M.G.); zuzana.dankulincova@upjs.sk (Z.D.V.); 2 Olomouc University Social Health Institute, Palacky University in Olomouc, Univerzitni 22, Olomouc 771 11, Czech Republic; 3 Institute of Research on Children, Youth and Family, Masaryk University, Jostova 218/10, Brno 602 00, Czech Republic

**Keywords:** adolescence, youth subcultures, excessive Internet use, protective factors, Slovakia

## Abstract

Youth subculture affiliation (SA) appears to be an important risk factor with regard to adolescents’ problem behavior. Excessive Internet use (EIU) has emerged as a new type of problem behavior; however, it has not yet been studied in adolescents affiliated with youth subcultures. Thus, the aim of this study was to assess the association between SA and EIU and to explore the role of selected protective factors. We used data from the Health Behaviour in School-aged Children (HBSC) study conducted in 2014 in Slovakia. The final sample for this study comprised 532 adolescents (mean age: 15.4; 49.6% boys). Hierarchical linear regression analysis was conducted to examine the associations of EIU with SA. Adolescents with SA were more likely to report EIU. Adjustment for protective factors decreased the association between EIU and SA. From all tested interactions, only the interaction of SA with family support was found to be significant. The relationship between family support and EIU was mediated via Monitoring by the mother only in adolescents without SA. Our findings imply that the risk of EIU is higher in adolescents with SA. There was a difference in how protective factors worked in adolescents with and without SA.

## 1. Introduction

Youth subcultures are dynamic entities characterized by specific lifestyle, music preference, distinctive world view, and shared values and behaviors of young people [1,2]. Terms, such as youth culture, neo-tribe, lifestyle and/or scene, are often used in the available literature to capture the postmodern nature of this phenomenon [2,3,4]. We will adhere to the term “youth subculture” as one of the appropriate options. According to the Health Behaviour in School-aged Children (HBSC) study performed in Slovakia in 2014, approximately 60% of adolescents are affiliated with one of the most prevalent subcultures, namely Hip-Hop/Rap (30%), Techno (12%), Metal (4%), Punk (2%) or with multiple subcultures (10%) [5].

Youth subculture affiliation (SA) appears to be an important risk factor with regard to adolescents’ problem behaviors, such as substance use, fighting, bullying, truancy, and early sexual behavior [6]. Those types of problem behaviors, which are often highly correlated, may be manifestations of the same underlying psycho-social factors. Occurrence of one type of problem behavior can predict other problem behaviors, and vice versa, and at the same time these are often associated with the same set of psycho-social determinants [7,8,9].

Given the proliferation of technology and new media into the everyday life of adolescents, Excessive Internet Use (EIU) has emerged as a new type of risk behavior. Time spent on the Internet itself is not a good criterion for assessing potentially pathological behavioral patterns [10]. Internet use is considered to be problematic when it shows signs of addiction, such as preoccupation, tolerance (spending more time on the Internet to achieve the same satisfaction), repeated efforts to reduce or stop, negative mood effects (irritability, depression) when Internet use is limited, staying online longer than intended, negative impact on work or relationships, lying about Internet use and using the Internet as a way to regulate mood [11]. Results of the international study EU Kids Online II, covering a representative sample of more than 18,000 adolescents from 25 European countries, suggest that only 5% of respondents showed moderate (4.4%) or high (1.4%) levels of EIU [12]. It might be that addiction does not develop on the Internet as such. As pointed out by Griffiths (2018), the Internet is just a medium for engaging in addictive behavior, such as online gaming, shopping, or social networking [10]. Nevertheless, prior research on this topic has reported that adolescents reporting EIU had higher instances of several problems, such as poor mental health [13], low self-esteem [13], unhealthy lifestyle [14] or substance use [15,16]. Although the etiological mechanisms contributing to the onset of Internet-related problems are to this date unclear, the strong association between EIU and other problem behaviors is evident. As previous studies showed a clustering of problem behaviors [7,8,9], it might be expected that EIU belongs to one of those clusters. This assumption is reflected in the hypotheses that adolescents affiliated with youth subcultures might be at higher risk of this type of behavior.

The role of protective factors in the adoption and persistence of problem behaviors has been shown in many previous studies. Family, as an important source of these protective factors, was shown to be associated with lower rates of different forms of problem behavior, e.g., substance use, aggressive behavior or school-related outcomes in adolescents with, as well as without, SA [17,18]. Moreover, a recent review of longitudinal studies on EIU identified family factors as one of the main predictors associated with EIU [19].

Thus, the aim of this study was to assess the association between SA and EIU and to determine whether a lack of parental monitoring, parental rules and family support account for these associations.

## 2. Materials and Methods

### 2.1. Sample and Procedure

We used data from the HBSC study conducted in 2014 in Slovakia. The study was conducted in accordance with the Declaration of Helsinki, and the protocol was approved by the Ethics Committee of the Medical Faculty at Safarik University in Kosice (NO: 9/2012). Parents were informed about the study via the school administration and were given the opportunity to opt out if they desired. Participation in the study was fully voluntary and anonymous, with no explicit incentives provided for participation. Questionnaires were administered by trained research assistants in the absence of a teacher during regular class time.

To obtain a representative sample, a clustered sampling was used. In the first phase, 151 elementary schools of various sizes located in rural, as well as in urban, areas from all regions of Slovakia were asked to participate. These were randomly selected from a list of all eligible schools (all primary schools with the required 5th to 9th grades) in Slovakia obtained from the Slovak Institute of Information and Prognosis for Education. We used data from the 130 schools that agreed to participate (86.1%). A total of 10,179 adolescents from the 5th to 9th grades of these schools (response rate: 78.8%) participated in the study. Following the International protocol of the HBSC study, the dataset was sent to the Data Bank Manager in Bergen in order to filter out non-valid cases and outliers within the required age structure (only 11-, 13- and 15-year-olds were included for further analyses). The cleaned dataset comprised 1596 11-year-old adolescents, 2200 13-year-old adolescents and 1449 15-year-old adolescents. More detailed information on the study design can be found elsewhere [20].

The final sample for this study consisted of adolescents who responded to questionnaires that included measures on EIU and SA. These measures were used only among 15-year-old adolescents (*N* = 1449) in 50% of the questionnaires administered (*N* = 683). Two versions of the questionnaire, with different sets of optional measures, were created in order to cover all the topics of national interest and, at the same time, to make administration within regular class time possible taking the length of the questionnaire into account. In order to ascertain the representativeness of this sub-sample, random selection was used for the distribution of these two versions of the questionnaire within a class. As a result, the final sample comprised 683 adolescents, of which 532 (mean age: 15.4; 49.6% boys) responded to the two essential questions for this manuscript (SA and EIU).

### 2.2. Measures

Excessive Internet use (EIU) of adolescents was measured by an EIU scale [21], which consisted of 5 items with 4-point Likert-type responses and covered the 5 dimensions of behavioural addiction: salience (I have gone without eating and sleeping because of the Internet); withdrawal symptoms (I have felt bothered when I cannot be on the Internet); tolerance (I have caught myself surfing when I am not really interested); relapse (I have tried unsuccessfully to spend less time on the Internet); and conflict (I have spent less time than I should with either family, friends, or doing schoolwork because of the time I spend on the Internet) [22]. Responses ranged from “never/almost never” (1) to “very often” (4). The EIU scale was created as the sum score of the five items (Cronbach’s alpha = 0.81).

Time spent on digital devices was measured by asking adolescents a single question about the amount of time they spent using electronic devices, such as computers, tablets or smart phones for emailing, tweeting, Facebook, chatting, surfing the Internet, etc. [23]. Responses were on an 8-point scale and ranged from “none at all, half an hour a day” (0) to “7 h or more a day” (7).

“Family well off” was measured by asking adolescents a single question about their perception of how well off they think their family is [24]. Responses ranged from “not at all well off” (1) to “very well off” (5).

Parental monitoring was measured by asking adolescents about their perception of what their mother and father knew about their activities and whereabouts (5 items) [23,24]. Responses ranged from “don’t have or don’t see mother/father” (0) to “she/he knows a lot” (4). Cronbach’s alpha was 0.83 for mothers and 0.92 for fathers.

Family support was measured using the Perceived Social Support Scale (PSSS), which is a 12-item self-reported questionnaire assessing perceived social support in three dimensions (from family, friends and significant others) [25]. For the purpose of our study, we used the subscale for family (4 items). A 7-point Likert-type format was used, ranging from “totally disagree” to “totally agree” (Cronbach’s alpha = 0.92).

Subculture affiliation (SA) was measured by asking adolescents a single question about whether they would classify themselves as being affiliated with one of the listed subcultures [6]. Possible responses were: Hip-hop/Punk/Skinheads/Techno/Metal/Church community/Other/I would not classify myself as affiliated with any subculture*.* Those who classified themselves as affiliated with a deviance-prone subculture (Hip-hop, Punk, Skinheads, Techno, Metal) were categorized as “adolescents with a SA.” The rest of the sample (Church community, Other and No affiliation) was categorized as “adolescents without a SA.”

### 2.3. Statistical Analyses

After the description of the sample, hierarchical linear regression analysis was conducted to examine the associations of EIU with SA, subsequently adjusted for gender, family well off and computer use (Model 1), parental monitoring (Model 2), and family support (Model 3) and interactions of SA with monitoring by the mother and family support (Model 4). Furthermore, we tested the mediation effect of monitoring by mother on the association between family support and EIU separately for adolescents with and without SA using Sobel tests. All data were analyzed using IBM SPSS statistics 21.0 (IBM Corporation, New York, NY, USA) for Windows.

## 3. Results

Based on descriptive statistics (Table 1), statistically significant differences between adolescents with and without SA were found regarding EIU, the amount of time they spent on a computer and monitoring by the mother. No statistically significant differences were found regarding family well off, monitoring by the father and family support.

Adolescents with SA were more likely to report EIU (Table 2, Model 1). Each of the adjustments for protective factors further decreased the association between EIU and SA to a less significant level (Table 2). Monitoring by the mother (Table 2, Model 2) and family support (Table 2, Model 3) were significantly and negatively associated with EIU, but no significant association was found regarding monitoring by the father (Table 2, Model 2). When family support was entered into Model 3, the association between monitoring by the mother and EIU decreased to a non-significant level. From all the tested interactions, only the interaction of SA with family support was found to be significant (Table 2, Model 4). According to the Sobel tests, the relationship between family support and EIU was mediated via monitoring by the mother only in adolescents without SA (Table 1).

## 4. Discussion

This study assessed the association between (SA) and (EIU) to determine whether a lack of parental monitoring, parental rules and family support account for these associations. SA was strongly and significantly associated with EIU, and adjustment for protective factors decreased the strength of this association. Based on significant interaction of SA with Family support, subsequent Sobel tests revealed that the relationship between Family support and EIU was mediated by Monitoring by the mother, but only in adolescents without SA.

Our findings regarding the significant association between SA and EIU are in line with cluster behavior theory seeing that EIU, like other types of problematic behavior, might be the manifestation of specific underlying individual, social, or environmental factors [26]. Similarly, a recent review of longitudinal studies on EIU identified the main predictors associated with EIU, namely, psychopathology, academic disposition, personal attributes and the influence of contextual factors, such as family context, peer context and the classroom environment [19]. Regarding personal attributes in this context, Griffiths (2000) highlighted the role of predisposition to behavioral addictions, which may contribute to the onset of several types of problem behaviors [27]. More concretely, a self-regulation deficit may play a substantial role in the occurrence of several problem behaviors [28].

We also aimed to determine whether a lack of protective factors (parental monitoring and family support) account for the association between SA and EIU. Although adjustment for lack of protective factors decreased the strength of the association between SA and EIU, this association remained statistically significant. This is in line with previous research on other types of problematic behaviors in youth subcultures, which showed that although protective factors do affect problem behaviors to some extent, being affiliated with youth subcultures is a much stronger factor in adolescents [29].

The significant interaction between SA and family support suggests that there is a difference between adolescents with and without SA in how social support influences EIU. Family support seems to be a significant protective factor decreasing EIU only in adolescents with SA. In adolescents without SA this effect is mediated by monitoring by the mother. In contrast, monitoring by the mother does not seem to serve as a protective factor regarding EIU in adolescents with SA. It is likely that adolescents affiliated with youth subcultures are those who are rather hard to monitor [29]; thus, it might not be that their parents are not trying, but that it is simply not working for these adolescents the same way it works in adolescents without SA. Although considerable evidence suggests that family factors, such as parental monitoring, may have a protective effect on the onset of problematic Internet use among the general adolescent population [30], the influence of peers seems to be more important in youth with SA [31]. Peer group identification was found to be associated with the emergence of multiple risk behaviors, such as substance use or aggressive behavior [32]. In line with this, the onset and occurrence of several types of risk behaviors (including EIU) among the group of adolescents with SA is affected more significantly by peers compared to parental influence.

The strength of our study is that it comprises relevant data from a representative sample of adolescents. EIU is quite a new phenomenon that has not been previously studied in adolescents with SA. A limitation of our study could be that we used a cross-sectional design, which did not allow us to explore causal pathways. Using self-reported data on problem behavior might also be considered a limitation of our study, although it has been previously shown to offer satisfying reliability [33]. Furthermore, a substantial amount of cases were omitted from final analyses which might suggest that representativeness of the sample and generalizability of our results is limited. However, the standard procedures included in the protocol of HBSC study guarantee the representativeness of the subsample comprising of 15-year-old adolescents. The two groups of 15-year-old adolescents who filled in the two partially different sets of questionnaires were selected randomly and do not differ in their main characteristics. Finally, we explored possible differences in family characteristics in those who did not fill in questions on SA and EIU compared to those who answered all of the questions interesting us. No statistically significant differences were found. The factors that protect adolescents from EIU despite their SA deserve further study, preferably using a longitudinal design and triangulation in order to explore causal pathways and verify the information from various sources instead of relying solely on self-reported data.

## 5. Conclusions

Our findings imply that the risk of EIU is higher in adolescents affiliated with youth subcultures. There is a difference in how protective factors work in adolescents with and without SA. In adolescents with SA, family support is the factor that seems to matter. In adolescents without SA the protective effect of family support is mediated by monitoring by the mother.

Stimulating specific parenting skills which serve as protective factors might be effective not only with regard to EIU, but also regarding other problem behaviors which seem to accumulate in youth subcultures. However, there is still a substantial number of adolescents with SA that do not use the Internet in an excessive way; moreover, they do not behave riskily at all. Exploring protective factors, which strengthen the resilience of adolescents with SA might be important. Further research in this area should be carried out with a longitudinal design in order to identify causal pathways.

## Figures and Tables

**Table 1:** Descriptive statistics by subculture affiliation (SA).

	Total	Adolescents without SA		Adolescents with SA		*p*-Value
	*N* = 580	*N* = 226	(%)	*N* = 354	(%)	
**Gender**						<0.01
Boys	264	91	41.6	173	55.3	>0.05
Girls	268	128	58.4	140	44.7	
	***N***	**Mean score (SD)**		**Mean score (SD)**		
**EIU**	532	7.62 (2.43)		8.65 (3.48)		<0.001
**Time spent on DD ^a^**	527	4.28 (2.00)		4.93 (2.32)		<0.001
**Family well off**	526	3.78 (0.86)		3.78 (0.86)		>0.05
**Parental monitoring**						
Mother	513	13.11 (2.52)		12.48 (2.69)		<0.01
Father	519	10.72 (4.45)		10.62 (3.76)		>0.05
**Family support**	514	23.50 (5.19)		23.18 (5.48)		>0.05

^a^ Digital Devices (DD) in hours; EIU: excessive internet use.

**Table 2:** Linear regression between excessive Internet use (EIU) and subculture affiliation (SA) subsequently adjusted for gender, family well off and computer use (Model 1), parental monitoring (Model 2), and family support (Model 3) and interactions of SA with monitoring by mother and family support (Model 4).

EIU	Model 1	Model 2	Model 3	Model 4
	Beta^sig^	Beta^sig^	Beta^sig^	Beta^sig^
Gender	−0.017	0.001	−0.029	−0.031
Family well off	−0.141 **	−0.126 **	−0.081	−0.080
Time spent on DD ^a^	0.160 ***	0.139 **	0.151 ***	0.164 ***
SA	0.125 **	0.117 **	0.111 *	0.615 *
Monitoring mother		−0.123 **	−0.067	−0.127
Monitoring father		−0.041	−0.025	−0.024
Family support			−0.232 ***	−0.024
SA × Monitoring mother				0.204
SA × Family support				−0.746 ***

^a^ Digital Devices (DD) in hours; * *p* < 0.05, ** *p* < 0.01, *** *p* < 0.001.
